# Supplementary material for: Cell Cycle Sensing Shapes Human T Cell Fate and Exhaustion Programs
Source: bioRxiv. 2026 Jun 12:2026.06.11.731737. Preprint. [Version 1] doi: 10.64898/2026.06.11.731737 (PMC13277874; doi:10.64898/2026.06.11.731737)
Supplement: Supplementary file 1 [file NIHPP2026.06.11.731737v1-supplement-1.pdf]

bioRxiv preprint doi: https://doi.org/10.64898/2026.06.11.731737; this version posted June 12, 2026. The copyright holder for this preprint (which was not certified by peer review) is the author/funder, who has granted bioRxiv a license to display the preprint in perpetuity. It is made available under a CC-BY 4.0 International license.

## Supplemental figures

Amouzgar et al. 2026

bioRxiv preprint doi: https://doi.org/10.64898/2026.06.11.731737; this version posted June 12, 2026. The copyright holder for this preprint (which was not certified by peer review) is the author/funder, who has granted bioRxiv a license to display the preprint in perpetuity. It is made available under a CC-BY 4.0 International license.

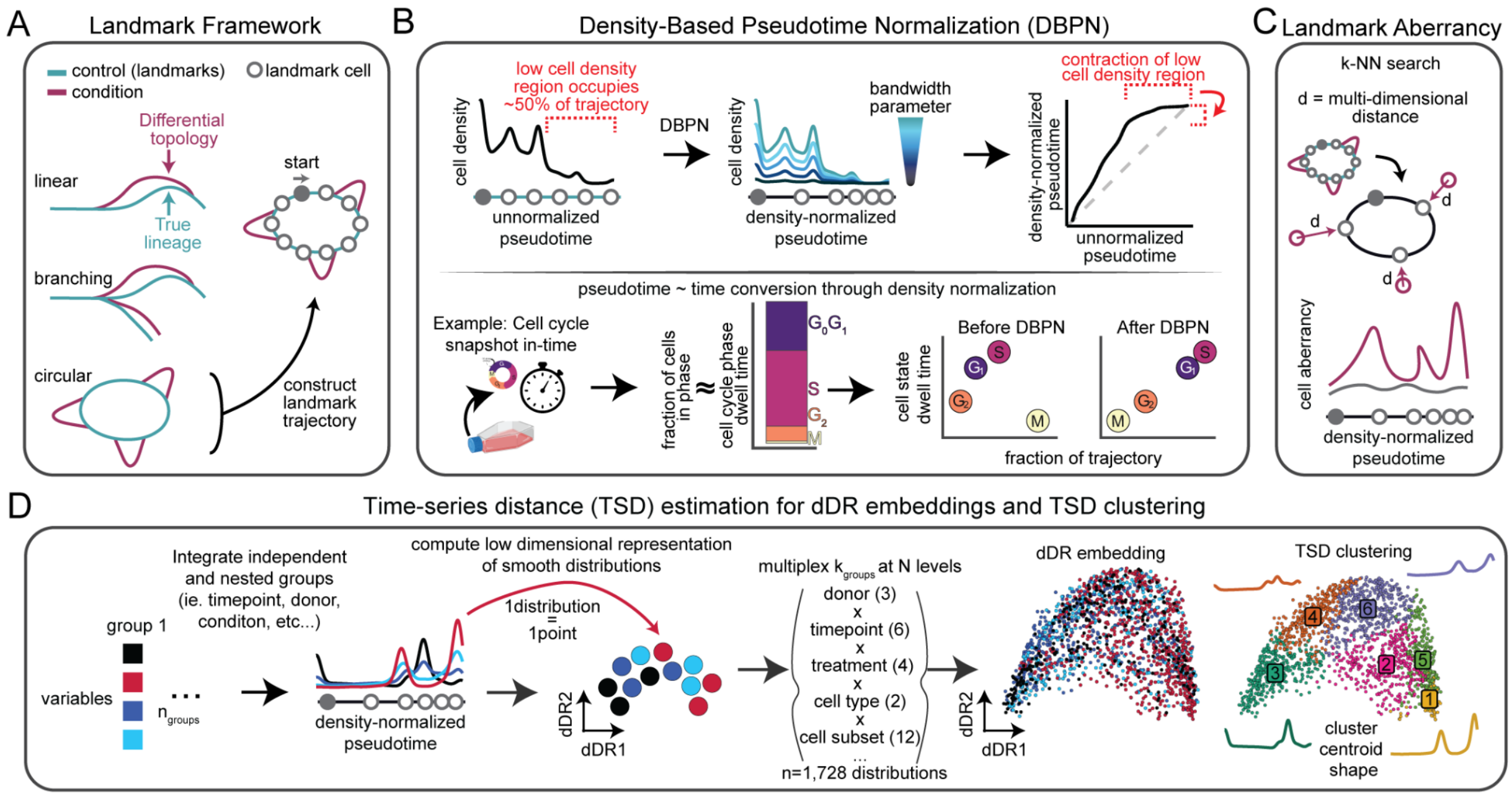


***Supplementary Figure 1. Hafez: a cell cycle landmark trajectory inference and time-series analysis framework generalizable to other systems***

***(A)*** *Schematic of traditional single-cell lineage topologies and an example of a landmark-based trajectory inference strategy for cyclical topologies using the Hafez framework.* ***(B)*** *Cell density-based pseudotime normalization (DBPN) that expands and contracts pseudotime estimates (left). Pseudotime-time conversion after DBPN matches trajectory fraction with dwell time of cell states in a dynamic system (right).* ***(C)*** *Schematic of example landmark aberrancy analysis in Hafez using Euclidean distance of nearest neighbor landmark cells.* ***(D)*** *Schematic of Hafez's time-series distance (TSD) estimation across different groups of interest for visualization using dDR$^{Hafez}$ (dynamic time warping Dimensionality Reduction) embeddings and TSD clustering$^{Hafez}$. Supplemental Figure 1 includes additional examples.*

bioRxiv preprint doi: https://doi.org/10.64898/2026.06.11.731737; this version posted June 12, 2026. The copyright holder for this preprint (which was not certified by peer review) is the author/funder, who has granted bioRxiv a license to display the preprint in perpetuity. It is made available under a CC-BY 4.0 International license.

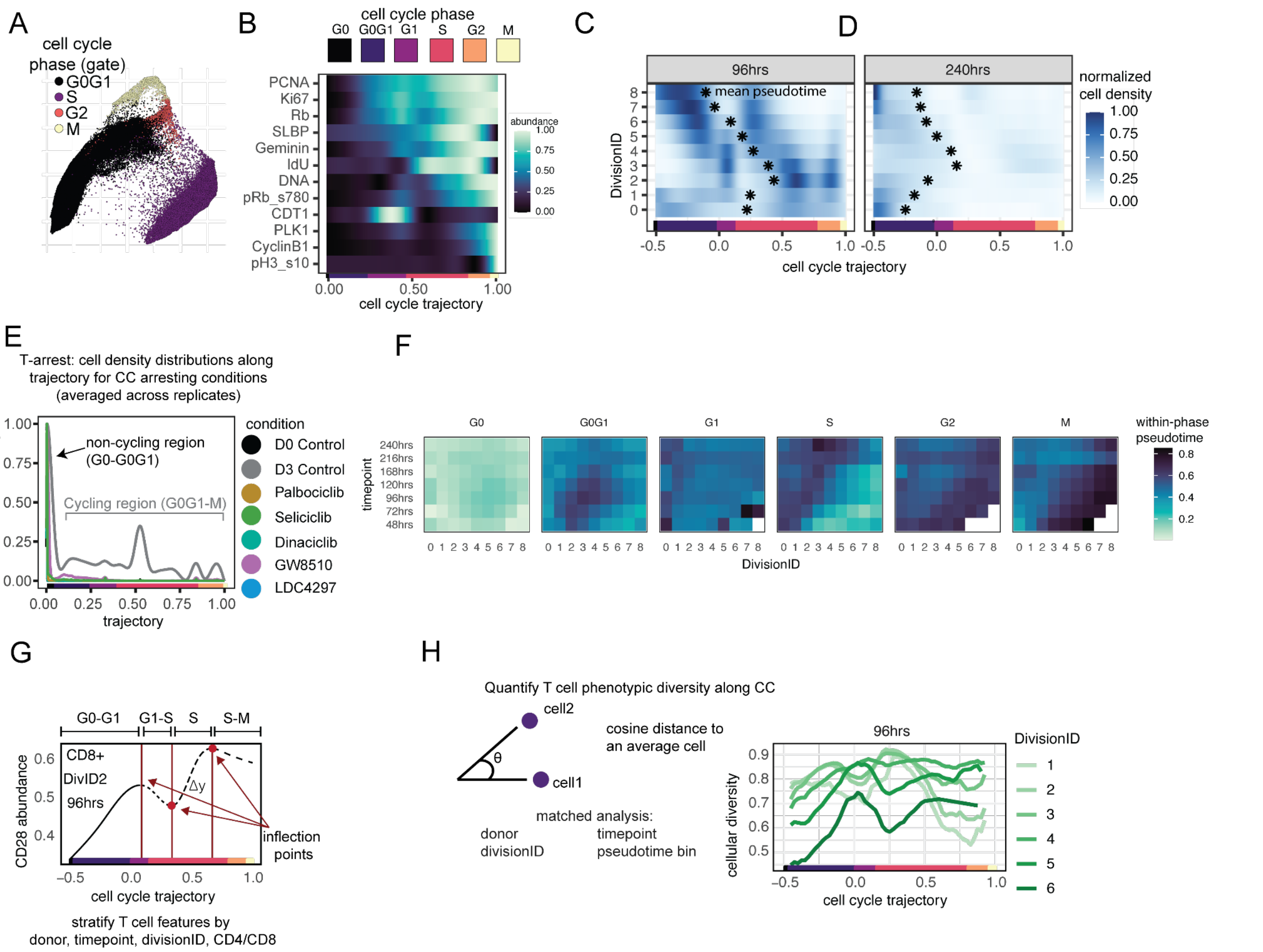


***Supplementary Figure 2: Cell cycle over time and division in primary human T cells***
***(A)*** *T cell CC embedding across all donors colored with manually gated CC phases.* ***(B)*** *Molecular abundance of CC molecules along CC trajectory.* ***(C)*** *CC pseudotime across divisions at 96hrs and* ***(D)*** *240 hrs, with points indicating mean pseudotime* ***(E)*** *Average cell density distributions along a cell cycle trajectory for each treatment group (D0 Control, n=1. D3 Control, n =3. Palbociclib, n=3. Seliciclib, n=3. Dinaciclib, n=3. GW8510, n=1. LDC4297, n=3).* ***(F)*** *Averaged CC cell density across all donors over time and division.* ***(F)*** *Average CC pseudotime across time and division normalized between 0 and 1 for each trajectory-annotated CC phase to highlight phases with potential  time- or division-dependent changes in cell distributions.* ***(G)*** *Depiction of CD28 molecular abundance along a cell cycle trajectory with example inflection points.* ***(H)*** *Cosine similarity over divisions at 96hrs.*

bioRxiv preprint doi: https://doi.org/10.64898/2026.06.11.731737; this version posted June 12, 2026. The copyright holder for this preprint (which was not certified by peer review) is the author/funder, who has granted bioRxiv a license to display the preprint in perpetuity. It is made available under a CC-BY 4.0 International license.

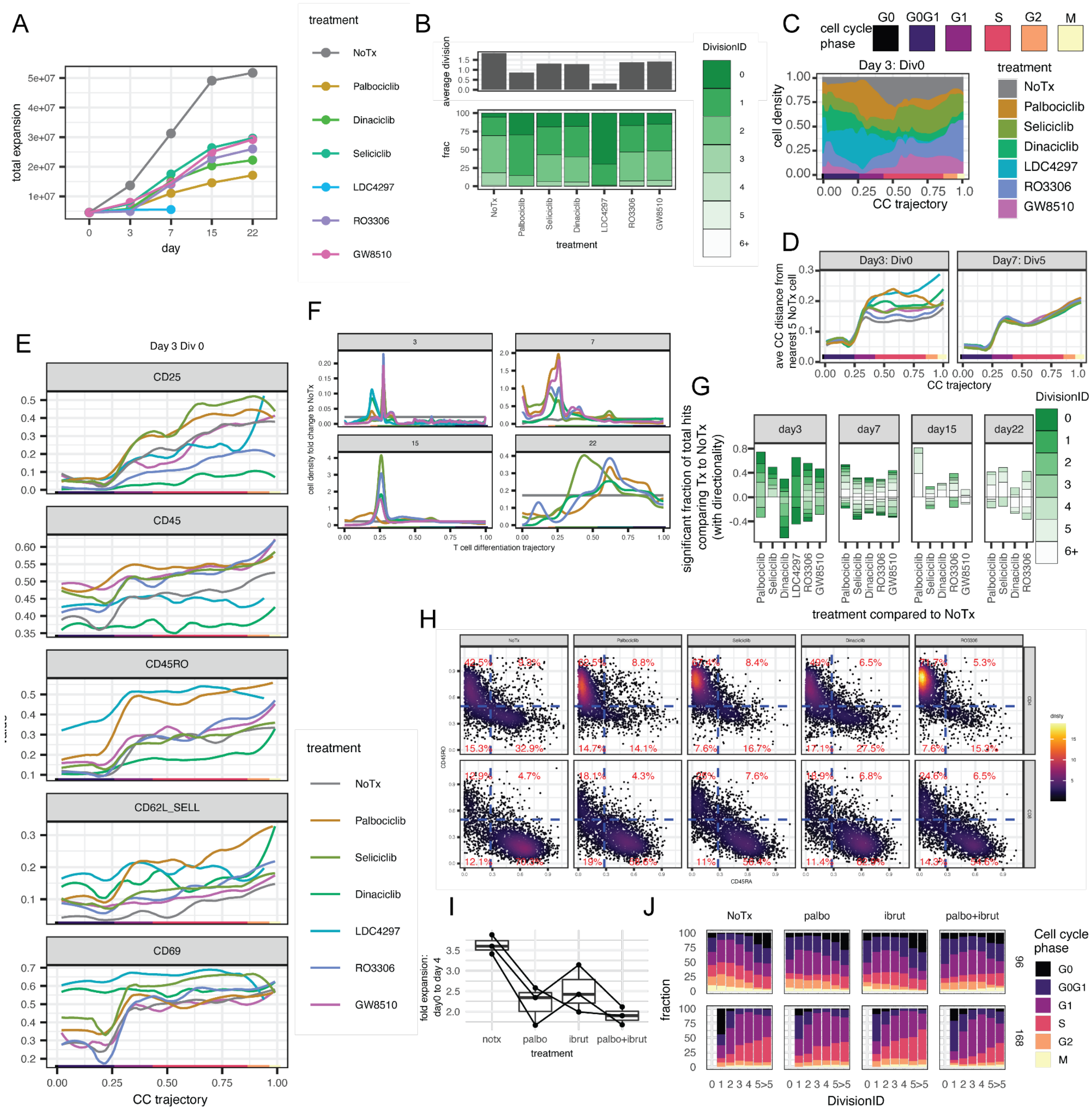


**Supplementary Figure 3: CDK inhibition and ITK blockade**

***(A)*** *Viable cell expansion counts for each treatment over time. All drugs slowed reduced expansion over time. Cells treated with LDC4297 (inhibiting CDK7) did not survive after Day 7.* ***(B)*** *Average division based on CFSE annotations and the fraction of cells in each DIvision ID for day 3 post-stimulation. All drugs observed fewer divisions with treatment.* ***(C)*** *Normalized T cell density of all conditions along the CC trajectory constructed on untreated T cells. We observe more CC skewing towards G0, G0G1, and G1 states in undivided cells on day 3 compared to non-treatment.* ***(D)*** *NN aberrancy score computed using the Hafez framework. Drug-induced, early transient CC aberrancies in undivided cells on day 3 of drug washout resolved in highly divided cells by day 7.* ***(E)*** *Protein abundance of example markers for day 3, undivided cells.* ***(F)*** *Fold change in cell density for each treatment compared to Control within each timepoint.* ***(G)*** *Significant fractions summarized by Division IDs.* ***(H)*** *CD45RO vs CD45RA biaxial plot of day 22 cells with >5 divisions from each treatment split by CD4 and CD8 cells.* ***(I)*** *Fold expansion from*

bioRxiv preprint doi: https://doi.org/10.64898/2026.06.11.731737; this version posted June 12, 2026. The copyright holder for this preprint (which was not certified by peer review) is the author/funder, who has granted bioRxiv a license to display the preprint in perpetuity. It is made available under a CC-BY 4.0 International license.

*day 0 to day 4 for each treatment group.* ***(J)*** *Trajectory-based CC annotations for each treatment over Division at 96hrs and 168hrs.*

bioRxiv preprint doi: https://doi.org/10.64898/2026.06.11.731737; this version posted June 12, 2026. The copyright holder for this preprint (which was not certified by peer review) is the author/funder, who has granted bioRxiv a license to display the preprint in perpetuity. It is made available under a CC-BY 4.0 International license.

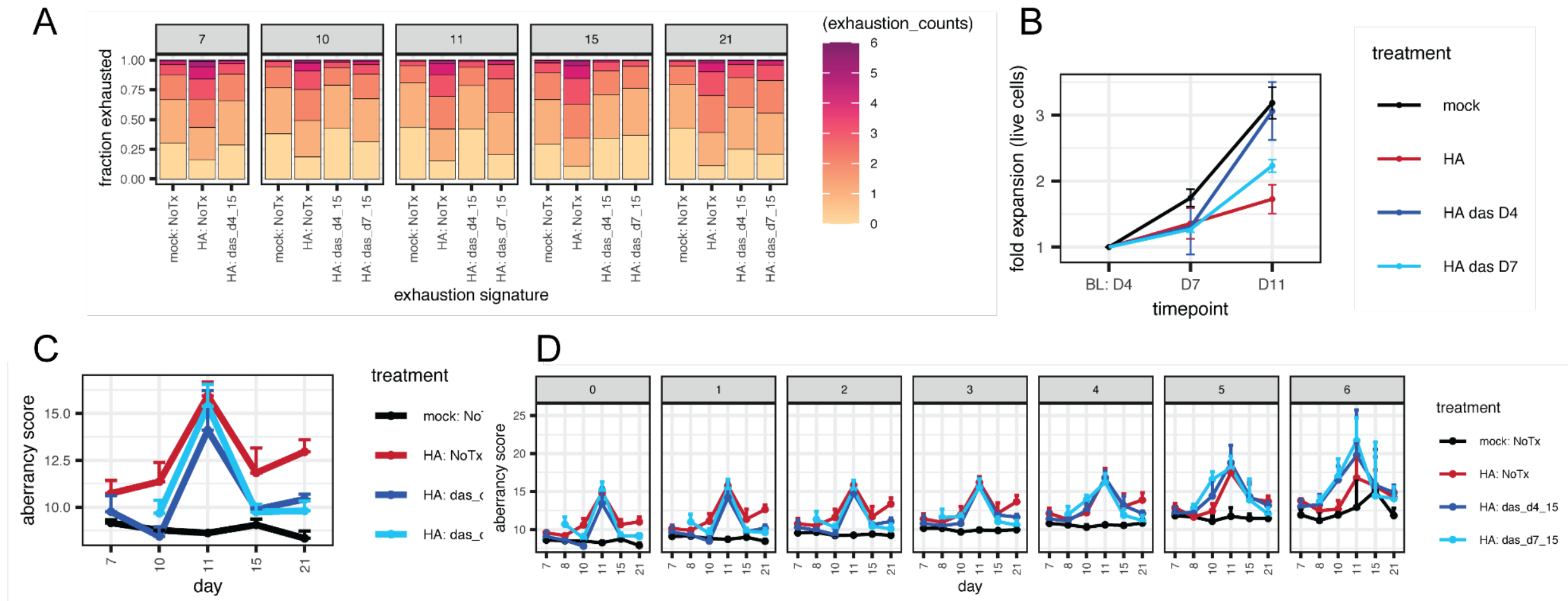


***Supplementary Figure 4: CC aberrancy analysis in Mock and HA CAR T cells treated with receptor signaling present or absent with dasatinib treatment***

***(A)*** *Fraction of cells with exhaustion signatures.* ***(B)*** *CC aberrancy over time.* ***(C)*** *CC aberrancy over stratified by exhaustion score.* ***(D)*** *Differential features on each day for HA CAR T cell groups without and with dasatinib treatment compared to mock matched by donor and exhaustion signature.*

bioRxiv preprint doi: https://doi.org/10.64898/2026.06.11.731737; this version posted June 12, 2026. The copyright holder for this preprint (which was not certified by peer review) is the author/funder, who has granted bioRxiv a license to display the preprint in perpetuity. It is made available under a CC-BY 4.0 International license.

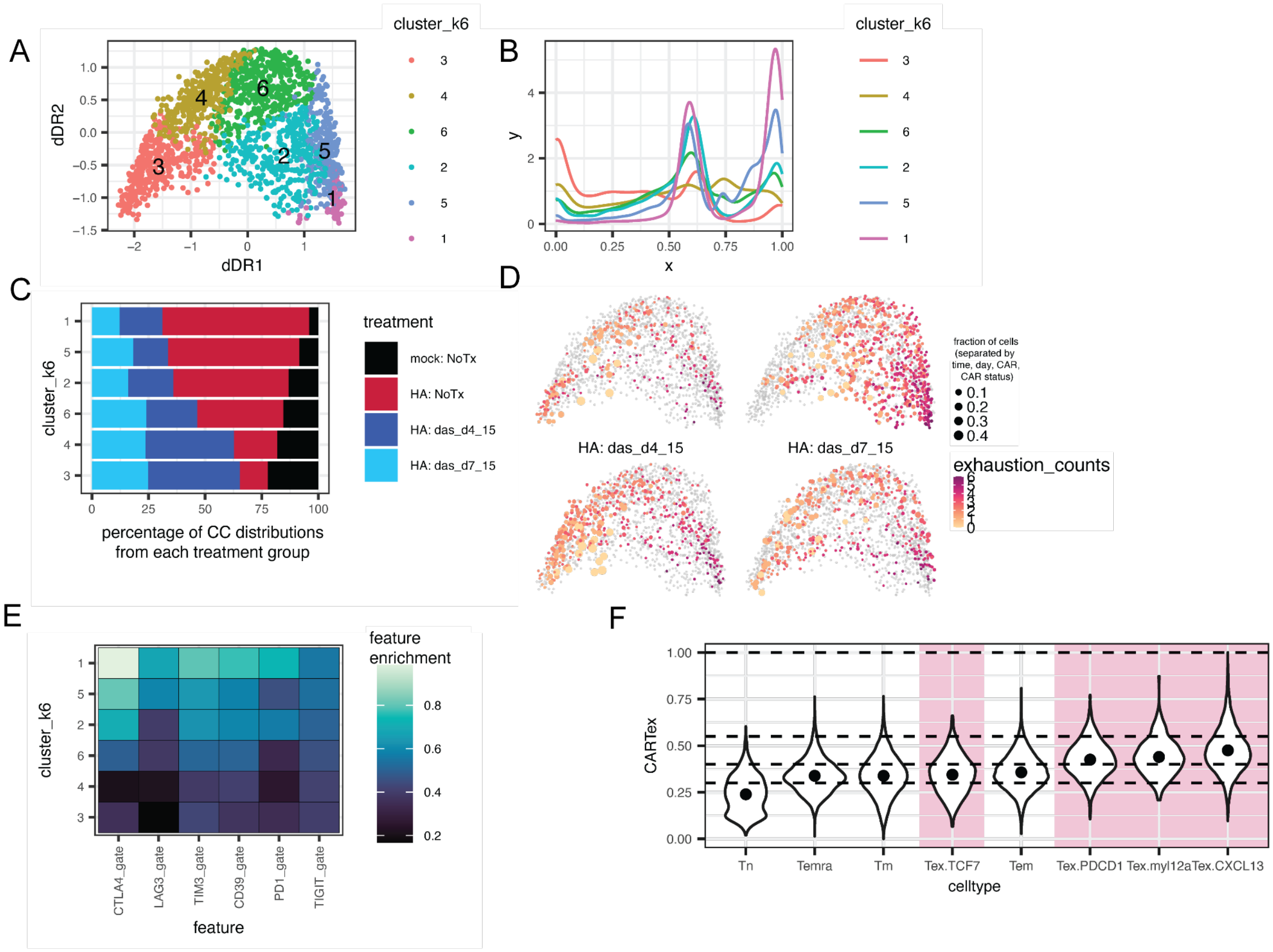


***Supplementary Figure 5: CC aberrancy in tonic signaling models of exhaustion and exhaustion scores in pan-cancer CD8 TILs***

***(A)*** TSD *clusters of CC cell density patterns.* ***(B)*** TSD *clusters patterns.* ***(C)*** *Abundance of treatment groups in each* TSD *clusters (fractions).* ***(D)*** *dDR$^{Hafez}$ embedding, with point size corresponding to the fraction of cells with each exhaustion signature.* ***(E)*** *Enrichment scores for each exhaustion marker in each cluster.* ***(F)*** *CARTex signature and CARTex bins for each celltype in the pan-cancer CD8 TILs dataset.*
